# Interplay between membranes and biomolecular condensates in the regulation of membrane-associated cellular processes

**DOI:** 10.1038/s12276-024-01337-5

**Published:** 2024-11-01

**Authors:** Nari Kim, Hyeri Yun, Hojin Lee, Joo-Yeon Yoo

**Affiliations:** Department of Life Sciences, Pohang University of Science and Technology, Pohang, Republic of Korea

**Keywords:** Cell biology, Molecular biology

## Abstract

Liquid‒liquid phase separation (LLPS) has emerged as a key mechanism for organizing cellular spaces independent of membranes. Biomolecular condensates, which assemble through LLPS, exhibit distinctive liquid droplet-like behavior and can exchange constituents with their surroundings. The regulation of condensate phases, including transitions from a liquid state to gel or irreversible aggregates, is important for their physiological functions and for controlling pathological progression, as observed in neurodegenerative diseases and cancer. While early studies on biomolecular condensates focused primarily on those in fluidic environments such as the cytosol, recent discoveries have revealed their existence in close proximity to, on, or even comprising membranes. The aim of this review is to provide an overview of the properties of membrane-associated condensates in a cellular context and their biological functions in relation to membranes.

## Liquid droplet behavior of membrane-associated biomolecular condensates explored

Understanding how cells organize and compartmentalize their interiors is key to understanding how they function and respond to changes. This study analyzes data from past research to explore the roles of membrane-associated biomolecular condensates in cellular processes such as signaling, membrane dynamics, and the formation of specialized cellular domains. Researchers demonstrate how these condensates, formed through a process called liquid–liquid phase separation, selectively gather specific molecules while excluding others, thus regulating various cellular functions. The study also investigates how these condensates influence membrane shape, contribute to contact between different organelles, and control the movement of molecules within the cell. Researchers conclude that a deeper understanding of these condensates offers new insights into cell biology and may lead to new treatment strategies for diseases in which condensate function is disrupted. This summary was initially drafted using artificial intelligence, then revised and fact-checked by the author.

## Introduction

Liquid‒liquid phase separation (LLPS) is a demixing phenomenon of macromolecules in solution driven by multiple weak and transient interactions^1,2^. As early as the 19th century, the cytoplasm was proposed to contain suspended droplets with diverse chemical properties^3^. Since the first illustration of the liquid-like behaviors of P granules within the embryo of *Caenorhabditis elegans*^4^, LLPS has emerged as one of the principles governing the compartmentalization of cellular spaces without membranes. Various types of biomolecular condensates, LLPS-driven structures composed of multivalent scaffolding macromolecules of proteins and nucleic acids, have been reported in the cytoplasm (e.g., stress granules, p-bodies)^5^ and nucleoplasm (e.g., nuclear speckles, nucleoli)^6^. These condensates play diverse roles, including regulating reaction kinetics^7^, signaling cascades^8^, selective sequestration of components^9^, assembly of granules^10,11^, and even modulation of gene expression^12,13^.

Although initially proposed as a mechanism for compartmentalizing macromolecules without membranes, the assembly of biomolecular condensates proximal to, or even tethered to, membranes has emerged. Since the pioneering demonstration of the LLPS behavior of the adhesion receptor Nephrin at the plasma membrane, along with its cytosolic partners Nck and N-WASP^14^, diverse types of membrane-associated molecular condensates have been reported. These condensates are found not only at the plasma membrane^8,15–17^, but also at various organelle membranes, including the endoplasmic reticulum (ER)^18–22^, nuclear envelope^23^, peroxisome^24^, and autophagosome^25,26^. Membrane-associated condensates restricted to particular cell types have also been reported to contribute to specialized cellular properties, as exemplified at tight junctions in epithelial cells^16^ and synapses in neurons^27,28^.

This review focuses on biomolecular condensates associated with membranes, providing an outline of their cellular and functional characteristics in terms of the regulation of membrane dynamics, morphology, organization, signaling, and even organelle tethering (Table 1). Specifically, we cover two types of membrane‒associated biomolecular condensates: membrane‒*anchored* and membrane-*bound* condensates. When a scaffold protein essential for phase separation is a membrane protein, biomolecular condensates are formed on the membrane, referred to as membrane-*anchored* condensates; in contrast, those that are recruited to membranes are referred to as membrane-*bound* condensates.

**Table 1:** Membrane-associated biomolecular condensates and their functions in cells.

Organelle	Name	Phase separation scaffold protein (in vitro)^a^	Category	Molecular mechanism	Membrane association^b^	Refs.
PM	Lat cluster	pLAT/Grb2/Sos1	Promote TCR signal transduction	Recruit kinase ZAP70, Exclude phosphatase CD45	Anchored	^8^
				Increase dwell time of Sos1 on membrane		^45^
	Nephrin cluster	nephrin/Nck/N-WASP	Promote downstream actin assembly	Increase dwell time of N-WASP on membrane	Anchored	^14,15^
	FGFR2 condensates	p-EGFR2/SHP2	Promote downstream signaling	Recruit PLCγ1 and increase enzymatic activity	Anchored	^17^
	ZO assembly	ZO1, ZO2, or ZO3	Tight junction formation	Partitioning of tight junction proteins	Bound	^16^
	Wnt signalosome	Dvl2 or Axin1	Promote β-catenin stabilization signaling	Recruit Axin1 into signalosome and disrupt destruction complex	Bound	^43,44^
	Integrin cluster	FAK or p130Cas	Promote integrin clustering	FAK or p130Cas phase separation increase adhesion sites	Bound	^88^
	PSD condensates	PSD-95/GKAP/Shank3/Homer3	Promote downstream actin assembly	Recruit GTPase SynGAP and exclude gephrin	Bound	^27,42,75^
	Cavin1 assembly	Cavin1	Membrane remodeling in caveola	Electrostatic interaction- and IDR- mediated membrane deformation	Bound	^52^
	Endophilin cluster	Endophilin	Induce membrane adhesion and support budding necks	Multivalent interactions between endophilin and recruited LPD existed on lipid layer	-	^55^
	Eps15-Fcho1/2 assembly	Eps15 or Fcho1	Endocytic vesicle formation	Exchange of components in clathrin-mediated endocytosis	Bound	^54^
	Active zone	RIM, RIM-BP, ELKS	Cluster ion channels and synaptic vesicles	Cluster the cytoplasmic tails of VGCCs near the vesicle release sites	Bound	^28^
ER	STING condensates	STING	Suppress downstream signaling	Inhibits translocation of STING from ER to Golgi	Anchored	^18^
	FIP200 puncta	-	Autophagosomal membrane formation	Transient Ca^2+^ induce FIP200 puncta and specify autophagy initiation site	-	^58^
	Sec16A condensates	-	ER exit site organization	Partitioning of ERES components	-	^20^
	TIS granule	TIS11B	Increase surface protein expression	Transfer SET from the AU-rich mRNAs to the protein	Bound	^19,66^
	AGO condensates	dmAGO1, hsAGO2	Protein quality control	Promote ubiquitination and proteasomal degradation of nascent peptides	Bound	^67^
	Sec body	-	Inhibit COPII vesicle formation	Partitioning ERES components under stress	-	^69,70^
	SCOTIN condensates	SCOTIN	Inhibit COPII vesicle formation	Hijack Sec31-sec13 from other COPII complex	Anchored	^21^
			Inhibit spontaneous autophagy	Block contact between phagophore and ERES		^89^
ER/endosome			Organelle membrane contact	Homotypic interaction at the membrane contact sites		^59^
Nuclear envelope	LEM2 coat	LEM2	Nuclear envelop reformation	Making O-ring sealing between NE and microtubule	Anchored	^23^
Vacuole/IM	PAS (yeast)	Atg13/Atg17/Atg29/Atg31	Autophagosomal membrane formation	IM formation around PAS	Bound	^56^
Autophagosome	p62 droplet	p62/polyUb	Autophagosomal membrane formation	Membrane wetting	Bound	^25,90,91^
Peroxisome	Pex13 condensates (yeast)	Pex13	Peroxisome cargo import	Channel formation with Pex14 and import Pex5-cargo via phase separation	Anchored	^24^

^a^Phase separation scaffold protein (in vitro): Essential protein(s) are identified for phase separation in vitro. (-) indicates that the purified protein was not assessed for phase separation in vitro.

^b^Membrane association: When scaffold protein(s) essential for phase separation are membrane proteins, condensates are specified as “anchored”, and those that are recruited to membranes are specified as “bound”. (-) indicates that the membrane association mechanism is not yet clear enough to determine whether it is anchored or bound.

## Main text

### Membranes affect the thermodynamics of biomolecular condensates

While the assembly or progression of membrane-anchored condensates occurs on the surface of two-dimensional membrane spaces, their thermodynamics follow principles similar to those of three-dimensional phase separation^29,30^. During phase separation, macromolecules such as proteins and RNA are partitioned from their surrounding milieu and form a separate phase when the local concentration exceeds the critical concentration^29^. This process involves multivalent and promiscuous interactions, including van der Waals interactions, electrostatic interactions, hydrophobic interactions, and cation–π interactions^31^. Phase-separating proteins also present typical features, such as repetitive interaction motifs (e.g., SH3-proline rich motifs)^32^ or intrinsically disordered regions (IDRs)^33^.

Several parameters influence the thermodynamics of phase separation, including the concentration of macromolecules and physical factors such as temperature, salt, pH, and volume^34^. However, compared with proteins in solution, the diffusion of proteins on the membrane is restricted, leading to a lower threshold concentration for phase separation and smaller condensate sizes. This property is well demonstrated by Whi3, a glutamine-rich protein that forms ribonucleoprotein condensates on the ER membrane. When reconstituted in vitro, membrane association with Whi3 significantly reduces the particle diffusion rate, interfering with liquid droplet fusion and resulting in the formation of small condensates^22^. A similar result was reported for SCOTIN condensates, where the sizes of the membrane-anchored condensates were smaller than those of their membrane-free counterparts in cells^21^. In addition, in in vitro studies of pLAT phase separation, which is composed of pLAT, Grab2, and Sos1, phase separation of membrane-anchored pLAT was readily observable in a significantly lower concentration range of Grb2 and Sos1 (nanomolar concentration) than phase separation in solution (micromolar concentration)^30^.

The lipid-binding domains of proteins also play a role in liquid droplet formation on membranes. The oligomeric complex of SLP65 and CIN85, which is essential for B-cell activation^35^, couples with lipid vesicles and undergoes tripartite phase separation to form liquid droplets^36^. The N-terminal lipid-binding moiety of SLP65 works to reduce the concentration threshold for tripartite phase separation and limits droplet size in vitro. Additionally, during T-cell activation, the formation of cytoplasmic condensates that associate with and stabilize cholesterol-rich condensed membrane domains has also been observed^37^. These raft-like lipid domains act as nucleation sites, supporting the formation and stability of pLAT clusters. Disruption of lipid domains via inhibitors of sphingolipid and cholesterol synthesis blocks phase separation, indicating the functional significance of coupling lipid and protein condensates in T-cell activation^37^.

Cellular lipids, which are integral components of membranes, also affect condensation. For example, anionic phospholipids catalytically influence FUS condensates in vitro^38^. In bacterial FtsZ condensates induced by membrane-interacting SlmA in a cell mimic system^39,40^, encapsulating FtsZ with phospholipids triggers condensate formation at the membrane boundary, even under noncondensing conditions^40^.

### Membrane-associated molecular condensates function to control signal transduction

Receptors on membranes have been observed to organize into assemblies at the micrometer or submicrometer scale, exhibiting signaling activities that deviate from those of classical chain reactions^41^. Recent studies indicate that some of these receptor clusters are formed via phase separation, which is essential for efficient signal transduction. Several mechanisms illustrate how membrane-associated phase separation facilitates signal transduction.

#### Selective compartmentalization of signaling activators and inhibitors

**Fig. 1:**
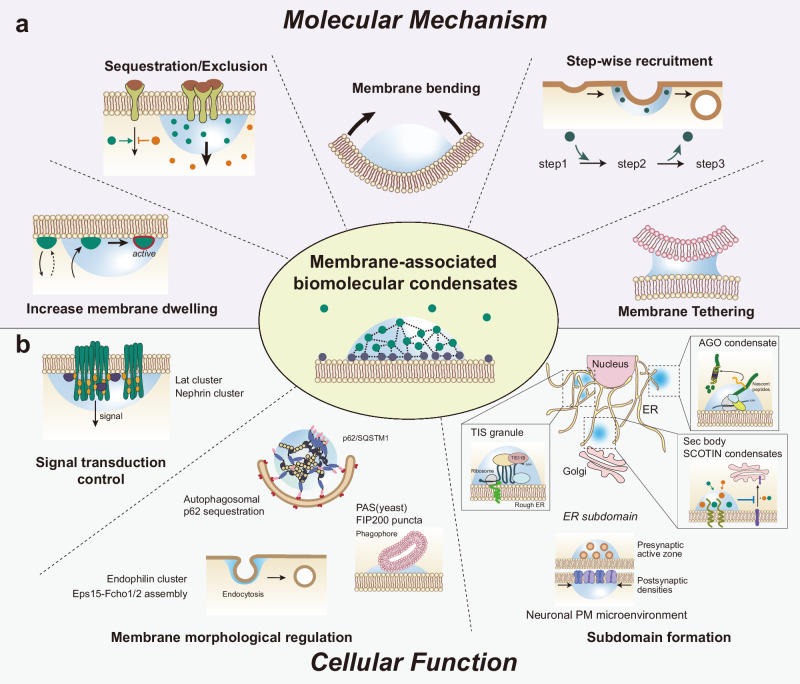
Biomolecular condensates are associated with cellular membranes. Representative molecular mechanisms (**a**) and cellular functions (**b**) of membrane-associated biomolecular condensates are illustrated. **a** (top-left) Similar to LLPS in solution, membrane-associated biomolecular condensates selectively sequester or exclude their constituents. (bottom-left) Membrane-associated biomolecular condensates prolong the membrane dwelling time of their constituents compared with conditions without condensates. (middle) Phase separation on a membrane can induce membrane bending when the attractive forces within it are sufficiently strong. (top-right) The liquid-like nature of biomolecular condensates enables efficient molecule exchange, facilitating stepwise processing. (bottom-right) Assembly of condensates on different membranes can tether the membrane together. **b** (top-left) Phase separation of receptor proteins such as Lat or nephrin enhances signal transduction efficiency. (bottom-left) Semiliquid-like droplets, represented by p62 in mammals, are engulfed by autophagic membranes, with a membrane wetting model implicated in this process. Phase separation of autophagy-related proteins at the initiation site of the autophagosome, such as the pre-autophagosomal structure (PAS) on the vacuole membrane in yeast and FIP200 droplet-like puncta on the ER membrane, has been reported. Phase separation of the endocytic machinery, exemplified by Eps15-Fcho1/2 assembly in clathrin-mediated endocytosis (CME) and formation of endophilin clusters in fast endophilin-mediated endocytosis (FEME), is required for productive endocytosis. (Right) Subdomain formation; TIS granules compartmentalize the ER domain for the translation of certain mRNAs. Environmental perturbations induce the formation of Sec bodies or SCOTIN condensates, inhibiting COPII-dependent ER-to-Golgi transport. These condensates hijack COPII components from the ER exit site, thereby arresting protein transport to the Golgi. AGO condensates associate with the ER membrane, recruiting E3 ligases for ubiquitin-dependent degradation of unwanted protein products. Phase separation at presynaptic active zones and postsynaptic densities creates a microenvironment to facilitate efficient signaling and transmission in neurons.

Membrane-associated phase separation sequesters signaling activators or excludes signaling inhibitors, creating a favorable environment for downstream signal transduction (Fig. 1a). For example, upon TCR activation, proteins such as pLAT, Grb2, and Sos1 assemble into submicrometer-sized clusters with liquid-like properties at the plasma membrane^8^. These clusters selectively sequester the kinase ZAP70 while excluding the phosphatase CD45, enabling sustained LAT phosphorylation and efficient TCR signaling^8^ (Fig. 1b). Similarly, postsynaptic density (PSD) proteins (PSD-95, SAPAP1, Shank3, and Homer3) undergo LLPS, clustering the N-methyl-D-aspartate (NMDA) receptor to the membrane-bound condensates in vitro^42^. These condensates enrich synaptic proteins (e.g., SynGAP) and an enzyme (e.g., CaMKII) and facilitate actin bundle formation while selectively excluding inhibitory postsynaptic proteins such as gephyrin^42^. In receptor tyrosine kinase (RTK) signaling, pEGFR2/SHP2 undergoes LLPS at the plasma membrane upon ligand stimulation, recruiting PLCγ1 to form a stable ternary complex that activates signal transduction^17^. The formation of the Wnt signalosome is also mediated by membrane-bound Dvl (Dishevelled) condensates, which recruit Axin1 to the plasma membrane^43,44^, disrupting the β-catenin degradation complex and initiating signal transduction^44^.

#### Increased dwell time of signaling proteins

Membrane-associated phase separation can increase the dwell time of signaling proteins on the membrane following recruitment from the cytoplasm (Fig. 1a). Upon ligand recognition, numerous signaling proteins are recruited to the membrane, and their conformational changes to an activated form enable downstream signal transduction. For example, Sos1, a guanine nucleotide exchange factor (GEF) and scaffold protein within the LAT cluster, requires an extended membrane dwell time for effective downstream activation^45^. This process is facilitated by the phase separation of pLAT/Grb2/Sos1 on the membrane (Fig. 1b)^45^. Another example is found during actin assembly, where microsized Nephrin/Nck/N-WASP undergoes phase separation^14^. This condensate recruits the actin nucleation factor Arp2/3 complex, promoting actin assembly at the membrane site^15^. Clustering increases the dwell time of N-WASP and Arp2/3, influencing the rate of cellular actin assembly and contributing to signal-specific activation and noise reduction^15^ (Fig. 1b).

#### Regulation of signal-dependent membrane-to-membrane translocation

Membrane-associated condensates can control signal-dependent translocation required for downstream signaling. STING, an integral ER protein, is activated by cyclic GMP-AMP (cGAMP), which is produced after cytosolic DNA detection^46^. Upon activation, STING translocates from the ER to the Golgi apparatus, where it interacts with TBK1 and IRF3, leading to interferon expression^46^. However, prolonged activation results in the formation of STING condensates on the ER membrane, preventing its translocation to the Golgi^18^. These STING condensates include TBK1, but not IRF3, halting the signaling cascade for interferon production^18^. In this scenario, the cGAMP-STING-TBK1 condensate functions as a sponge, absorbing excess cellular cGAMP and modulating the cellular response to prevent overactivation^18^.

### Membrane-associated biomolecular condensates and membrane morphology

The shape of a membrane, which is influenced by its lipid composition or associated proteins, is closely linked to its functions^47^. Recent studies suggest that the assembly of membrane-associated condensates can induce morphological alterations in membranes. In in vitro systems, interactions between condensates and membranes, whether membrane-anchored or nonanchored, can induce membrane deformation^48^. This process is modulated by the compositions and properties of both the membranes and the condensates^48^. Early studies on synthetic phase separation driven by polymers encapsulated by membranes have shown that parameters influencing “wetting” affect membrane remodeling^49^. Recently, it was discovered that biomolecular condensates can also induce various membrane morphologies in a similar manner^50–52^. For example, when the low-complexity domain of FUS is artificially linked to a giant unilamellar vesicle (GUV), it generates protein-lined inward membrane tubules, indicating that phase separation exerts a potent physical force to induce changes in membrane curvature (Fig. 1a)^51^. The purified cavin1 protein undergoes LLPS in an IDR-dependent manner and induces membrane tubules from GUV in vitro^52^. IDR-dependent membrane remodeling by cavin1 is required for the formation of caveolae, cave-like membrane structures at invaginated membrane sites^52^. The autophagosomal membrane wraps the p62/SQSTM1 substrate through the wetting of p62 condensates on the membrane (Fig. 1b)^25^.

During endocytosis, the lipid bilayer reshapes, undergoes membrane bending, invagination, bottleneck formation, and scission^53^. Recent studies have emphasized the role of the liquidity of phase-separating complexes on the plasma membrane in the formation of endocytic vesicles. Clathrin-mediated endocytosis (CME) initiation factors, including the F-Bar domain only proteins 1 and 2 complex (FCHO1/2), adaptor protein AP2 complex, clathrin, and epidermal growth factor receptor substrate 15 (EPS15), coalesce into liquid-like clusters on the membrane through multivalent interactions^54^. This liquid-like property is necessary to generate productive endocytic structures in cells (Fig. 1b)^54^. Liquid–liquid phase separation gathers the components of the initiation complex at the endocytic site, and the rapid molecular exchange within the assembly allows the release of initiation components to proceed with the steps of endocytosis (Fig. 1a)^54^. Additionally, during fast endophilin-mediated endocytosis, endophilin and lamellipodin (LPD) form liquid-like clusters, which are involved in membrane adhesion and the generation of negative Gaussian curvature (Fig. 1a, b)^55^.

Similar to endocytosis, membrane-associated molecular condensates play a pivotal role in the initiation and nucleation steps of double-layered autophagosome biogenesis. In budding yeast, starvation induces the formation of the pre-autophagosomal structure (PAS), a liquid-like condensate composed of the Atg1 complex^56^. The PAS is bound to the vacuole membrane, where PAS maturation occurs via Atg1 autophosphorylation, followed by phagophore formation^56^ (Fig. 1b). In mammalian cells, autophagy-inducing stimuli recruit the ULK1 complex to the cytosolic surface of the ER membrane, initiating the formation of the phagophore from the ER^57^. FIP200, a component of the ULK1 complex, also forms fusion-prone liquid-like puncta upon autophagy induction^58^ (Fig. 1b).

### Molecular condensates associated with the membrane regulate organelle tethering

Membrane-enclosed organelles give rise to stable or transient regions of close apposition known as membrane contact sites (MCSs). Recent findings suggest that the homotypic interaction via the IDR of SCOTIN, which is capable of phase separation^21^, tethers the ER and endosome membranes (Fig. 1a), thereby controlling perinuclear endosome positioning for proper endosome trafficking^59^.

At MCSs, membrane tethering occurs through multiple protein interactions, many involving domains predicted to be IDRs^60^. The flexibility or extensibility of IDRs are crucial for regulating the tethering of organelle membranes^60,61^. For example, electrostatic interactions between the IDRs of ORP5 and ORP8 localize at MCSs between the ER and plasma membrane, functioning to tether these membranes^62^. VAP proteins, which are localized at the ER membrane, act as receptors for FFAT motif-containing proteins in other organelles to initiate MCS formation^63^. The extensible IDR of VAP-A is required for tethering the ER specifically with the Golgi but not with mitochondria^61^. It has not yet been reported whether the IDRs of MCS proteins other than SCOTIN can form condensates. Considering their unique protein composition and dynamic formation, investigating the potential of MCS proteins for phase-separating properties is worthwhile.

### Membrane-associated molecular condensates generate subdomains on the ER membrane

The ER comprises extensive cisternae and tubules, generating an interconnected network structure for protein and lipid synthesis, transport, and Ca^2+^ storage. Within the ER membrane, proteins and lipids are not uniformly distributed but are divided into distinct subdomains with consequential functions, as exemplified by rough ER or mitochondria-associated membranes^64,65^. Recent studies have shown that phase-separating assemblies occupy separate spaces on the ER membrane, resulting in functional subdomains.

#### TIS granules form the TIGER domain on the ER membrane

The RNA-binding protein TIS11B binds to AU-rich elements (AREs) within the 3’-UTR of mRNA, forming distinct TIS granules that intertwine with the ER subdomain known as the TIGER domain in cells^19^ (Fig. 1b). These TIS granules are assembled through the LLPS of TIS11B, driven by charged interactions between the positively charged N-terminus and the negatively charged C-terminus residues. TIS granules are enriched with ARE-rich mRNAs and ARE-binding chaperone proteins, enabling the translation of these mRNAs at the TIGER domain^19^. In contrast to the spherical shapes of membraneless organelles, TIS granules maintain mesh-like condensate structures across massive ER surfaces^19,66^. The formation of irregularly shaped TIS granules requires specific types of RNAs that are predicted to be largely disordered and form intermolecular RNA-RNA interacting networks^66^.

#### Protein quality control of AGO condensates on the ER membrane

Argonaute (AGO) undergoes phase separation on the ER and regulates both microRNA (miRNA)-mediated gene silencing and proteasome-dependent protein degradation^67^ (Fig. 1b). Generally, the AGO–miRNA interaction facilitates the efficient targeting of mRNAs, leading to mRNA repression and decay in the cytosol^68^. Argonaute associates with the ER membrane through a PI(4,5)P2 binding motif^67^. Furthermore, the assembly and disassembly of AGO condensates on the ER membrane are reversible, depending on their interaction with PI(4,5)P2^67^. ER-localized AGO condensates attract Ltn1, an E3 ubiquitin ligase, to ubiquitinate nascent peptides for the degradation of unwanted protein products^67^. This study suggests that by positioning AGO condensates on the ER membrane, the coupled regulation of posttranscriptional gene silencing and protein quality control can be achieved.

#### Condensates near ER exit sites (ERESs) inhibit ER-to-Golgi transport

ERESs are specialized subdomains of the ER where coat protein complex II (COPII)-coated vesicles emerge for the transport of secretory proteins and membrane cargo proteins. In *Drosophila* S2 cells, amino acid starvation inhibits cargo transport from the ER and triggers the formation of a liquid-like assembly containing ERES components, namely, the Sec body^69^ (Fig. 1b). Among the ERES components, Sec23 and Sec24A/B are key factors for Sec body assembly and function independent of their classical roles in COP II vesicle formation^69^. A fluorescence recovery after photobleaching (FRAP) analysis indicated that Sec bodies recover rapidly after bleaching, indicating that they possess liquid-like properties with dynamic molecular exchanges^69^. In the INS1 human insulin-producing cell line, amino acid starvation and sodium stress signals also induce the formation of Sec bodies, which are composed of the Sec16 and COP II subunits^70^. Mammalian Sec bodies exhibit reversible liquid-like properties similar to those of *Drosophila* Sec bodies. The formation of the Sec body acts as a rapid shutdown mechanism for the early secretory pathway during cellular stress, efficiently recovering once stress relief occurs.

Similarly, COPII-mediated transport is regulated by SCOTIN condensates. Interferon (IFN)-induced SCOTIN condensates hijack Sec31/Sec13 from other ERES components, blocking COPII-coated vesicle formation^21^ (Fig. 1b). Although the Sec body and SCOTIN condensates inhibit ER-to-Golgi transport by disrupting COPII assembly, the compositions differ. The Sec body includes all four components of COPII in addition to Sec16, while SCOTIN condensates sequester Sec31 and Sec13 but not Sec24 or Sec16^21,70^. Additionally, the kinetics of condensate assembly differ, as amino acid starvation induces Sec body formation within 4 h of treatment, whereas much longer IFN stimulation is required for SCOTIN condensate formation^21,70^.

### Membrane-associated molecular condensates establish mircoenvironments for controlling synaptic function

Neurons exhibit a high degree of morphological and functional polarization, with synapses serving as fundamental apparatuses for signaling and transmission across all components of the nervous system. Recent studies have provided compelling evidence suggesting that the development of compact postsynaptic densities (PSDs) and presynaptic active zones may be orchestrated through molecular assemblies mediated by phase separation processes^27,28,42^.

Postsynaptic densities are molecular assemblies composed of numerous proteins at extremely high concentrations. The sizes and molecular compositions of PSDs are continually adjusted in response to synaptic activities^71,72^. Excitatory postsynaptic densities (ePSDs) are constructed with a group of highly abundant multidomain scaffold proteins, including PSD-95, synapse-associated protein 90/postsynaptic density-95-associated protein (SAPAP), Shank, and Homer^42,73^. These proteins associate to create densely packed molecular condensates that are connected to, but not surrounded by, postsynaptic membranes^74^. The expansion or contraction of PSDs involves the addition or removal of proteins from PSD assembly, including glutamate receptors and their associated scaffolding proteins^71^.

SynGAP, a synaptic GTPase-activating protein, is abundant within the PSD and maintains a nearly stoichiometric ratio with PSD-95. The binding of SynGAP to PSD-95 induces phase separation, leading to the generation of densely concentrated, liquid-like droplets that resemble the PSD in vitro^27^ (Fig. 1b). Transmembrane AMPA receptor regulatory proteins (TARPs) also accumulate at the PSD and condense through phase separation. The condensation of TARPs into the PSD is driven by multivalent interactions between the C-terminal Arg-rich motif and PDZ12 of PSD-95^75^. These interactions between TARP and PSD-95 are necessary for AMPA synaptic transmission in the hippocampal neurons of mice^75^.

Like the assemblies found in the PSD, presynaptic active zones are self-organized and form compact protein network structures just beneath the plasma membrane^74^. They consist of various scaffold proteins, particularly those belonging to the UNC-10/RIM superfamily (including Piccolo and Bassoon), ELKS, SYD-2/liprin-α proteins, and RIM-BP^76^. These proteins establish interactions between the active zone and ion channels and MUNC-13, which engages with the machinery responsible for vesicle fusion^77^. The interaction between the three SH3 domains of RIM-BP and multiple proline-rich motifs, as well as various IDRs within RIM, drives the formation of condensates^28^. Through C-terminal-tail-mediated direct interactions with both RIM and RIM-BP, voltage-gated calcium channels (VGCCs) form condensates on supported lipid membrane bilayers through LLPS in vitro^28^ (Fig. 1b).

## Perspectives

Aberrant LLPS results in the formation of protein aggregates, a phenomenon strongly implicated in various neurological disorders. This is evidenced by the accumulation of β-amyloid, tau, α-synuclein, and TDP-43 in the brains of individuals with Alzheimer’s disease, Parkinson’s disease, and amyotrophic lateral sclerosis, respectively^78^. In the context of cancer, numerous instances of oncogenic mutations have been identified that dysregulate LLPS, leading to uncontrolled cell signaling and, in some cases, tumorigenesis^79^. In contrast to many dysregulated cytoplasmic or nuclear biomolecular condensates in disease^80–82^, relatively little is known about membrane-associated biomolecular condensates.

Amyotrophic lateral sclerosis-associated mutations in p62 result in condensates with reduced liquidity, leading to delayed autophagosomal degradation^83^. STING mutations with reduced condensation and enhanced IFN production have been reported in patients with autoimmune diseases^18^. Additionally, cancer-associated SCOTIN mutations have been linked to impaired condensation and ER-to-Golgi transport control^21^. Furthermore, recent comprehensive mapping analyses have indicated that mutations in focal adhesion and tight junction proteins might dysregulate condensation in autism patients^84^. Considering the accumulated cases of disease-associated mutations that dysregulate membrane-associated biomolecular condensates, future research should focus on validating the therapeutic potential of controlling these condensates.

One pioneering application utilizing the phase-separating properties of proteins has been the engineering of CAR-T cells. Here, IDRs from EWS, FUS, and TAF-15 are included to engineer T cells with enhanced membrane-proximal signaling and improved cytotoxicity against low-antigen-expressing cancers^85^. Additionally, Nck, a component of the nephrin cluster, is exploited by enteropathogenic *Escherichia coli* (EPEC) during invasion via the reorganization of actin filaments^86^. The ability of the synthetic peptide p1 to block the interaction of Nck and N-WASP inhibits nephrin cluster formation and prevents EPEC infection in cells^87^.

Compared with biomolecular condensates in solution, many technical challenges exist in the study of membrane-associated biomolecular condensates. First, the smaller size and irregular shapes of these condensates, along with their association with membranes, make it difficult to visualize the fine structures in cells. Second, the phase-separating properties of many membrane-associated condensates have been shown by artificially anchoring them to a lipid bilayer or GUV in vitro. However, to investigate the assembly dynamics of condensates composed of transmembrane proteins, more appropriate model systems that enable the study of the actual consequences of the embedded transmembrane proteins are needed.
